# Control of *Drosophila* Type I and Type II central brain neuroblast proliferation by bantam microRNA

**DOI:** 10.1242/dev.127209

**Published:** 2015-11-01

**Authors:** Ruifen Weng, Stephen M. Cohen

**Affiliations:** 1 Institute of Molecular and Cell Biology, 61 Biopolis Drive, Singapore 138673, Singapore; 2 Department of Cellular and Molecular Medicine, University of Copenhagen, Blegdamsvej 3, Copenhagen 2200 N, Denmark

**Keywords:** MicroRNA, Neural stem cell, Bantam, Prospero, Brat

## Abstract

Post-transcriptional regulation of stem cell self-renewal by microRNAs is emerging as an important mechanism controlling tissue homeostasis. Here, we provide evidence that bantam microRNA controls neuroblast number and proliferation in the *Drosophila* central brain. Bantam also supports proliferation of transit-amplifying intermediate neural progenitor cells in type II neuroblast lineages. The stem cell factors *brat* and *prospero* are identified as bantam targets acting on different aspects of these processes. Thus, bantam appears to act in multiple regulatory steps in the maintenance and proliferation of neuroblasts and their progeny to regulate growth of the central brain.

## Abstract (precis)

**Summary:** The *Drosophila* miRNA bantam regulates the expression of Brat and Prospero – known inhibitors of brain neuroblast proliferation – to modulate growth of the central brain.

## INTRODUCTION

In recent years, *Drosophila* neural stem cells have emerged as an important model for understanding stem cell function and regulation. The *Drosophila* larval central brain contains two types of stem cells, called neuroblasts (NBs). Type I NBs divide asymmetrically to self-renew and produce a daughter cell (called the ganglion mother cell, GMC), which divides only once to produce two terminally differentiated progeny (reviewed by Doe, 2008; Knoblich, 2008). Type II NBs divide asymmetrically to produce an intermediate neural progenitor (INP) cell that can undergo another four to eight rounds of additional asymmetric division, producing a GMC at each division (Bello et al., 2008; Boone and Doe, 2008; Bowman et al., 2008; Izergina et al., 2009). In having a transit-amplifying population of INP cells, the type II NB lineages in the *Drosophila* larval central brain more closely resemble mammalian neuronal stem cells (Merkle and Alvarez-Buylla, 2006). Understanding the homeostatic mechanisms that maintain ‘stem-ness’ and control proliferation will be important in understanding the roles of stem cells in tumorigenesis (Morrison and Kimble, 2006; Jiang and Reichert, 2014).

microRNAs (miRNAs) have been linked to regulatory feedback and feed-forward mechanisms, which suggests that they may serve as regulators of cellular homeostasis (Herranz and Cohen, 2010; Ebert and Sharp, 2012). A growing body of evidence indicates that miRNAs play an essential role in stem cells, in which cellular homeostasis is crucial for self-renewal and differentiation. Some miRNAs contribute to stem cell maintenance by repressing genes involved in differentiation (Gangaraju and Lin, 2009; Hattangadi et al., 2011; Yi and Fuchs, 2012; Shyh-Chang and Daley, 2013). In *Drosophila*, genetic analysis has linked miRNAs to regulation of stem cell maintenance and proliferation. Bantam miRNA has been implicated in maintenance of ovarian stem cells (Shcherbata et al., 2007). miR-124 activity is required to support proliferation of neuroblasts in the larval brain by limiting expression of Anachronism (Weng and Cohen, 2012). Target sites for miR-275 and miR-306 limit the expression of the differentiation factor Bag of Marbles in male germ line stem cells (Eun et al., 2013). *Drosophila* miR-305 acts on the Notch and Insulin signaling pathways in intestinal stems cells to place symmetric versus asymmetric stem cell division under nutritional control (Foronda et al., 2014). In mouse and human hematopoietic stem cells, the miR-99a/100∼125b miRNAs have been implicated in the regulation of stem and intermediate progenitor cell homeostasis by controlling the balance between TGFβ and Wnt signaling (Emmrich et al., 2014).

Development of the *Drosophila* central nervous system (CNS) relies, to a large extent, on control of neuroblast proliferation. In light of the roles of bantam miRNA in tissue growth control (Brennecke et al., 2003), in ovarian stem cells (Shcherbata et al., 2007) and in larval optic lobe (Li and Padgett, 2012), we sought to investigate whether bantam is required for the proliferation control of the central brain neural stem cells. *bantam* mutants have fewer neuroblasts and show a cell-autonomous effect on neuroblast growth and proliferation in the larval central brain, resulting in a reduction in the total number of post-mitotic neurons. We identify *brat* and *prospero* as functionally significant targets through which bantam controls type II neural progenitor growth and proliferation in the *Drosophila* brain. Evidence for a role of a third bantam target, the Notch pathway regulator *numb*, is equivocal.

## RESULTS AND DISCUSSION

### *bantam* is expressed in neural progenitors of the larval CNS

**Fig. 1.**
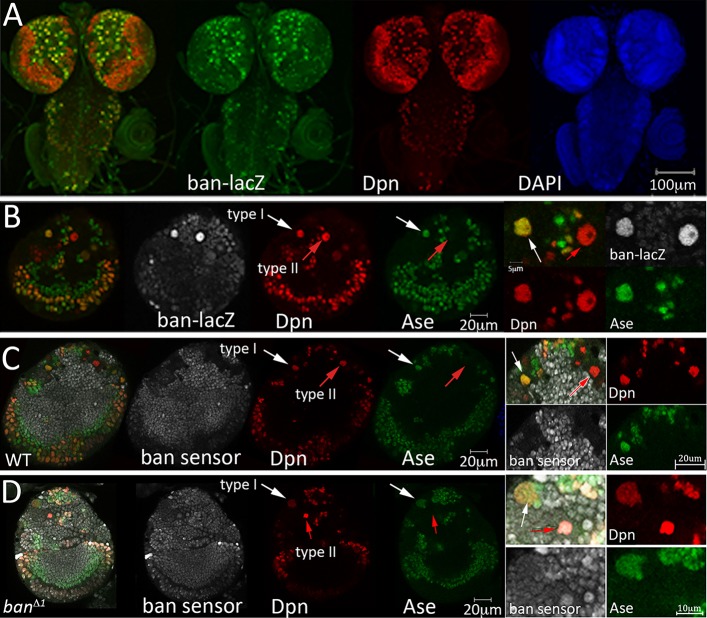
***bantam* expression in larval brain neuroblasts.** (A) Overview of *bantam-lacZ* expression (green) in the larval CNS, from a projection of optical sections at 20× magnification. Neural progenitors were labeled with anti-Dpn (red), DNA was labeled with DAPI (blue). (B) Single optical section showing *bantam-lacZ* expression (gray) in the central brain and optic lobes. Brains were labeled with anti-Dpn (red) and anti-Asense (Ase, green). White arrows indicate type I NB expressing Dpn and Ase; red arrows indicate type II NB expressing Dpn, but not Ase. Higher magnification views are shown on the right. (C,D) Single optical sections showing bantam GFP sensor expression (gray) in wild-type (WT) and *bantam^Δ1/Δ1^* homozygous mutant larval brains, labeled as in B.

As a first step to characterize the expression of *bantam* in brain neuroblasts, we examined a *lacZ* reporter transgene inserted at the *bantam* locus. In mature third instar larvae, *bantam-lacZ* expression was detected in the central brain, optic lobes and ventral nerve cord. High levels of *bantam-lacZ* were observed in large superficial cells that expressed the transcription factor Deadpan (Dpn), a neuroblast marker. Projection of a series of optical sections showed that *bantam-lacZ* was expressed in all Dpn^+^ cells (Fig. 1A), indicating that *bantam* is expressed in the neuronal progenitor cells of the larval central brain. *bantam-lacZ* was also expressed in the Dpn^+^ cells in the optic lobes, albeit at lower levels (Fig. S1).

Type I neuroblasts are characterized by nuclear expression of the transcription factors Dpn and Asense (Ase), and by cytoplasmic expression of the differentiation factor Prospero. Type II neuroblasts show nuclear Dpn expression, but do not express Asense or Prospero. *bantam*-*lacZ* expression was detected in type I (Dpn^+^Ase^+^) neuroblasts and in type II (Dpn^+^Ase^−^) neuroblasts (Fig. 1B). As an independent test for bantam activity, we made use of a sensor transgene that reports bantam activity through downregulation of a ubiquitously expressed GFP transcript containing bantam target sites in its 3′ UTR (Brennecke et al., 2003). In wild-type brains, sensor GFP was not detected in type I (Dpn^+^Ase^+^) or in type II (Dpn^+^Ase^−^) neuroblasts. GFP was also not detected in the cells immediately adjacent to the neuroblasts, which are most likely the GMCs or transit-amplifying INP cells, but GFP was seen in the many smaller cells that are probably their progeny (Fig. 1C). In the *bantam* mutant brain, GFP was detected in type I (Dpn^+^Ase^+^) neuroblasts and type II (Dpn^+^Ase^−^) neuroblasts, and in their GMC and INP daughters (Fig. 1D; additional examples in Fig. S2). These observations suggest that bantam is active in type I and type II neuroblasts and their immediate progeny, and that this activity is lower or absent in the differentiated progeny of these cells.

### *bantam* is required for larval CNS growth

**Fig. 2.**
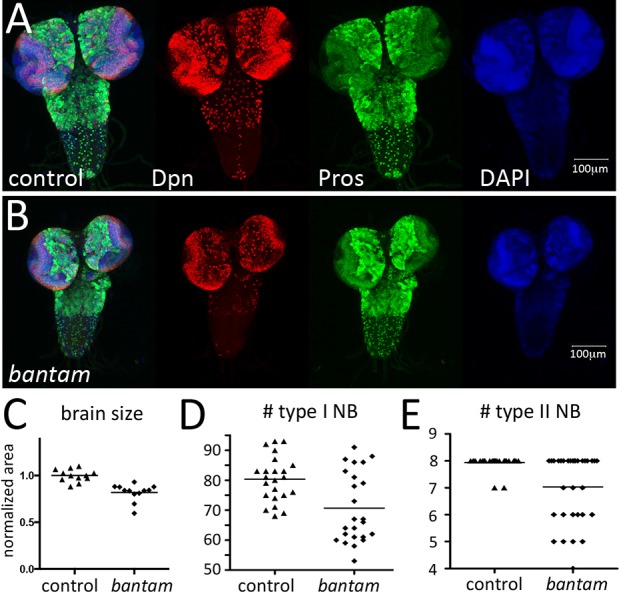
***bantam* is required for larval central brain growth.** (A,B) Overview of *bantam^Δ1^*^/+^ heterozygous control and *bantam^Δ1/Δ1^* homozygous mutant brains at late third instar larval stage. Neural progenitors were labeled with anti-Dpn (red), neural progeny were labeled with anti-Pros (green) and DNA was labeled with DAPI (blue). (C) Brain area was measured from confocal micrographs using ImageJ. Data for the mutant were normalized to the average of the control brains. *n*=14 brains/genotype. *P*<0.0001, Mann–Whitney test. (D,E) Number of type I and type II NB (per brain lobe) in *bantam^Δ1^*^/+^ (control) and *bantam^Δ1/Δ1^* homozygous mutants in late third instar. (D) Average=80 for control versus 71 for the mutant. *n*=24 brains/genotype. *P*=0.004, Mann–Whitney test. (E) Average=8 for control versus 7 for the mutant; *n*=32 brains/genotype; *P*=0.0002, Mann–Whitney test. Horizontal line: mean value.

Earlier studies on bantam showed a role in regulation of tissue growth and cell proliferation (Hipfner et al., 2002; Brennecke et al., 2003). Consistent with the overall reduction of body size in *bantam* mutants, the CNS was smaller in *bantam* mutants, with a ∼20% reduction in the central brain of animals homozygous for the *bantam^Δ1^* allele compared with control animals at the third instar larval stage (Fig. 2A-C). The average numbers of type I and type II neuroblasts were lower in *bantam^Δ1^* mutant brains (Fig. 2D,E). *bantam* has both cell-autonomous and systemic effects on tissue growth (Brennecke et al., 2003; Li and Padgett, 2012; Boulan et al., 2013; Huang et al., 2014). The latter are mediated via a repressive influence on ecdysone production. Given that ecdysone acts negatively on larval neuroblast proliferation (Homem et al., 2014), it is possible that the reduction in neuroblast number in *bantam^Δ1^* mutants could reflect a non-autonomous consequence of its regulation of ecdysone signaling. Alternatively, loss of neuroblasts could be a consequence of cell-autonomous effects of bantam on neuroblast growth and/or survival.

Type II neuroblasts produce INP cells that undergo several rounds of division forming multiple GMCs. The extended proliferative capacity of the type II INPs depends on the absence of Prospero from the INP nucleus: nuclear Prospero drives differentiation (Li and Vaessin, 2000; Choksi et al., 2006; Bayraktar et al., 2010). In type I GMCs, nuclear Prospero promotes differentiation. In type II INPs, Prospero is expressed, but remains cytoplasmic (Bayraktar et al., 2010). Prospero enters the nuclei of type II GMCs that are produced by the INPs to promote their differentiation, but remains absent from INP cell nuclei until their final division (Bayraktar et al., 2010).

**Fig. 3.**
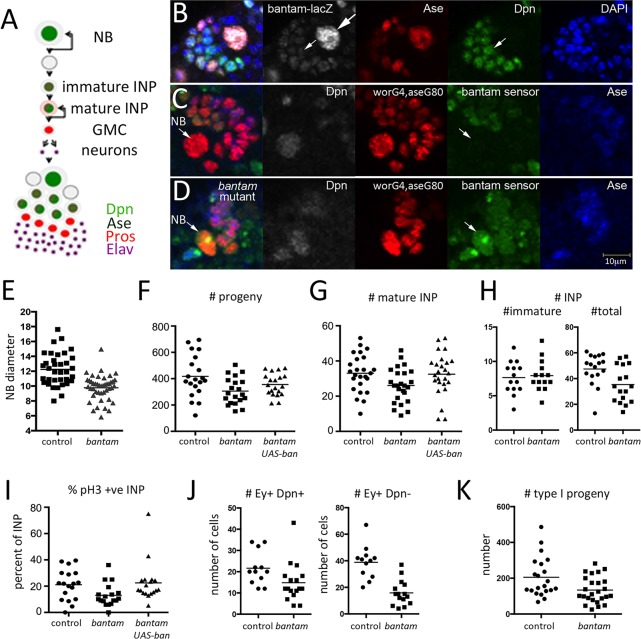
***bantam* is required cell-autonomously in type II NB lineages.** (A) Diagram representing a type II NB lineage. Cell types can be identified by a combination of molecular markers: type II NB: nuclear Dpn (light green); immature INP: nuclear Ase (dark green); mature INP: nuclear Dpn, nuclear Ase and cytoplasmic Pros (pink); GMC: nuclear Pros (red); post-mitotic neurons: nuclear Elav (purple). (B) *bantam-lacZ* expression (gray) in a single type II NB lineage labeled with anti-Dpn (green), anti-Ase (red) and DAPI (blue). Large arrow indicates the large Dpn^+^Ase^−^ type II NB. Small arrow indicates lower levels of *bantam-lacZ* observed in Dpn^+^Ase^−^ immature INP and in Dpn^+^Ase^+^ mature INP. (C) A single type II NB lineage showing bantam GFP sensor expression (green) in a WT control brain. The type II lineage was labeled by worniu-Gal4, asense-Gal80-driven UAS-RFP (red). Bantam sensor GFP (green) was very low in the Dpn^+^ (gray) Ase^−^ (blue) type II NB (arrow). (D) Bantam GFP sensor expression in a *bantam* mutant brain. Labeling as in panel B. Note the higher level of sensor (green) in the neuroblast and INPs due to loss of bantam-mediated repression of GFP expression. (E) Type II NB diameter (µm) in control (FRT2A) and *bantam^Δ1/Δ1^* mutant type II NB clones. Control: *n*=35 brains; *bantam^Δ1/Δ1^ n*=42 brains. Horizontal line: mean value. *P*<0.0001, Mann–Whitney test. (F) Total number of GFP^+^ progeny in control and *bantam^Δ1/Δ1^* mutant type II NB clones±*UAS*-*bantam*. *n*=20 clones/genotype. ANOVA: *P*<0.05. (G) Total number of mature INPs (Dpn^+^Ase^+^) in control and *bantam^Δ1/Δ1^* mutant type II NB clones±*UAS*-*bantam*. *n*=25/genotype. ANOVA: *P*<0.05. (H) Number of immature and total INPs in control and *bantam^Δ1/Δ1^* mutant type II NB clones. Immature INPs were identified by the absence of nuclear Dpn and presence of nuclear Ase in the MARCM clone. *n*=14/genotype. *P*=0.83 for immature INPs and *P*=0.01 for total INPs (Mann–Whitney test). (I) Percentage of mature INPs with anti-pH3 staining in control and *bantam^Δ1/Δ1^* mutant type II NB clones±*UAS*-*bantam*. *n*=18 clones/genotype. ANOVA: *P*<0.05. (J) Number of older INPs and their progeny in control and *bantam^Δ1/Δ1^* mutant type II NB clones. Older INPs were identified as cells in the MARCM clones expressing Deadpan and Eyeless (*P*=0.015, Mann–Whitney test). Progeny were identified as cells in the clones expressing Eyeless, but not Deadpan (*n*=12 control clones and 17 *bantam* mutant clones; *P*<0.0001, Mann–Whitney test). (K) Number of GFP-labeled progeny in type I lineages comparing control and *bantam^Δ1/Δ1^* mutant MARCM clones (*P*=0.017, Mann–Whitney test).

We were interested to examine how bantam activity is deployed in the more complex type II lineage. *bantam-lacZ* expression was highest in Dpn^+^Ase^−^ type II neuroblasts (Fig. 3B, large arrow). Lower levels were seen in the Dpn^−^Ase^+^ immature INP cells (Fig. 3B, small arrows) and in the more mature Dpn^+^Ase^+^ INP cells nearby. The pattern of bantam activity revealed by the bantam GFP sensor was consistent with the expression reported by the *bantam-lacZ* transgene. To visualize the neuroblast and its adjacent INP progeny, we made use of *worniu-Gal4*, which is expressed in type I and II neuroblasts, combined with *ase-Gal80* to repress Gal4 activity in the type I lineage. We used this combination to label cells within the type II lineages by driving *UAS-redStinger* expression. Bantam-sensor GFP levels were low in the Dpn^+^ neuroblast and in the adjacent INP cells, indicative of bantam activity. Sensor levels were higher in the more differentiated progeny of the lineage located deeper in the brain cortex, indicative of lower bantam activity. The difference in bantam-sensor levels between the proliferating cells and their differentiating progeny disappeared in the *bantam* mutant brain (Fig. 3D). Together, these observations indicate that *bantam* is expressed in the proliferating neuroblast and INP cells of the type II lineage.

### *bantam* is required cell-autonomously for neuroblast growth and proliferation

To investigate bantam function in the type II lineage in more detail, we compared genetically marked type II neuroblast clones with and without bantam activity using the mosaic analysis with a repressible cell marker (MARCM) strategy (Lee and Luo, 1999)*.* MARCM clones were induced in early first instar larvae and analyzed at 120 h after larval hatching (ALH). Clones derived from single wild-type or *bantam* mutant neuroblasts were compared for neuroblast growth and for the number of progeny produced. Although both control and *bantam* mutant clones contained only one large Dpn^+^Ase^−^ neuroblast per clone, the Dpn^+^ neuroblasts were smaller on average in *bantam* mutant type II clones than in the control clones (Fig. 3E). To determine the number of progeny produced by *bantam* and normal control neuroblasts, the number of GFP^+^ cells was counted (excluding the Dpn^+^ neuroblast). At 120 h ALH, *bantam* mutant clones contained fewer GFP-labeled small cells compared with the control clones or with mutant clones carrying a *UAS*-*bantam* transgene (Fig. 3F). As the total number of progeny reflects the proliferation of the type II neuroblast as well as its INP daughter cells, we sought to determine whether the number of INP daughters was affected. GFP-labeled cells that clustered around the type II neuroblast were identified as mature INPs if they expressed both nuclear Dpn and Ase. *bantam* mutant clones contained fewer mature INP compared with control clones or with mutant clones carrying a *UAS*-*bantam* transgene (Fig. 3G). The observation of fewer INPs in the clones could reflect the formation of fewer INP cells owing to reduced neuroblast division or a decrease in amplification of the pool by INP proliferation. We did not observe a significant decrease in the number of immature INPs in the *bantam* mutant type II clones (Fig. 3H; identified by the absence of nuclear Dpn and presence of nuclear Ase). The decrease in total INP number (Fig. 3H) probably reflects a reduction in INP proliferation in the *bantam* mutant clones. Consistent with this, we observed a decrease in the number of INPs labeled with the mitotic marker phosphohistone H3 in *bantam* mutant clones (Fig. 3I), providing evidence for reduced INP proliferation.

Bantam promotes cell growth and proliferation by limiting expression of negative growth regulators, as well as limiting apoptosis by repressing Hid expression (Brennecke et al., 2003; Herranz et al., 2012a,b). Therefore, a reduction in the number of INP daughter cells in *bantam* mutant clones could indicate increased cell death, as well as reduced proliferation. To determine whether the reduced number of mutant INPs was due to increased cell death, we expressed *UAS-Diap1* to block apoptosis in the *bantam* mutant MARCM clones. There was no significant difference in the average size of *bantam* mutant clones with or without Diap1 expression (Fig. S3). Together, these experiments provide evidence for reduced INP production by type II neuroblasts and reduced proliferative activity of these INPs.

To examine the consequences of reduced proliferation of the type II INPs, we looked at older INPs and their progeny, which express the transcription factor Eyeless (Bayraktar and Doe, 2013). There were fewer mature INPs expressing Eyeless and Deadpan (Fig. 3J) and fewer of their progeny (expressing Eyeless but not Deadpan; Fig. 3J; sample images in Fig. S4). This is consistent with a truncation of the type II lineage due to reduced proliferation of INPs in the *bantam* mutant. We also observed a reduction in the size of the type I lineages in *bantam* clones (Fig. 3K). It is likely that reduced proliferation in both types of neuroblast lineages contributes to the reduced size of the mutant brain (Fig. 2). To isolate the effect of type II lineage reduction, we used the *wor-Gal4, ase-Gal80* combination to direct expression of a bantam sponge in the type II lineage. This reduced overall brain size, but did not produce any obvious alteration in the gross morphology of the central brain (Fig. S5). We have not looked in detail for more subtle neuroanatomical consequences.

### Bantam regulates *prospero*, *brat* and *numb*

**Fig. 4.**
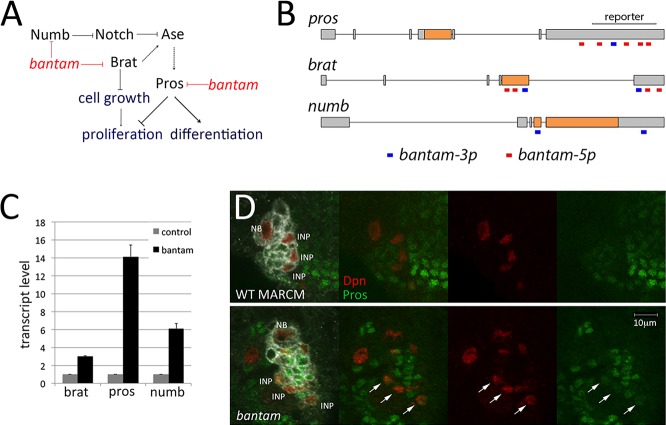
**Bantam targets multiple regulators of type II lineage progression.** (A) Diagram showing regulators of the type II lineage growth and proliferation, indicating potential bantam targets. (B) Diagram of the *prospero*, *brat* and *numb* transcripts showing predicted target sites for bantam-5p and bantam-3p. (C) Normalized *prospero*, *brat* and *numb* mRNA levels measured by quantitative PCR in RNA isolated from neuroblasts by TU tagging. Data show mean±s.d. of two independent biological replicates. (D) Prospero protein (green) in wild-type (WT) FRT2A and *bantam^Δ1/Δ1^* mutant type II NB clones. NBs and mature INPs were labeled with anti-Dpn (red). The clonal progeny are labeled white in the merged images. Arrows indicate INPs in the *bantam^Δ1/Δ1^* mutant clone expressing Dpn and nuclear Prospero.

Computational target prediction programs have not identified known regulators of neuroblast lineages as potential targets of bantam (e.g. www.targetscan.org). To allow for the possibility of target sites with atypical features, including GU base pairing to the miRNA seed sequence, we scanned the known regulators of type II lineage development using RNAHybrid (Rehmsmeier et al., 2004) and found potential sites for bantam in the *prospero*, *brat* and *numb* transcripts (Fig. 4A). The *bantam* primary transcript produces two mature miRNA products, processed from the two arms of the pre-miRNA hairpin. bantam-3p is considerably more abundant (www.mirbase.org) and is the form detected by the bantam-sensor transgene. We observed potential target sites for both the bantam-5p and bantam-3p miRNAs in the 3′ UTR of *prospero* and in both coding and 3′ UTR exons of *brat* (Fig. 4B). *numb* transcript contains predicted sites for bantam-3p (Fig. 4B)*.* The predicted pairing is shown in Fig. S6.

As a first step to determine whether any of these transcripts are regulated by bantam in neuroblast lineages, we examined mRNA levels by quantitative RT-PCR in RNA isolated using the TU tagging method (Miller et al., 2009). *insc*-*Gal4* was used to drive expression of *UAS-UPRT* in all neuroblast lineages in the larval central brain and newly synthesized RNA was labeled by TU incorporation. *prospero*, *numb* and *brat* levels each increased in TU-tagged RNA isolated from *bantam* mutant neuroblasts compared with that isolated from wild-type controls (Fig. 4C). Reciprocally, overexpression of *bantam* in neuroblasts with *insc*-*Gal4* driving a *UAS-bantam* transgene reduced the level of target gene expression to about half that of normal (Fig. S7). Together, these data provide evidence that bantam regulates *prospero*, *numb* and *brat* in central brain neuroblasts.

Despite the observed RNA increases in *bantam* mutant neuroblasts, we were unable to detect Brat or Numb proteins above background in these cells with the available antibodies. The increase in *prospero* transcript was larger in the *bantam* mutant, and we observed a change in Prospero protein expression in the mutant INP cells. Nuclear Prospero protein was not detectable in the Dpn^+^ INPs in control brains, but we observed low levels of Prospero together with Dpn in the nuclei of INPs in *bantam* mutant clones (Fig. 4D, arrows). Premature Prospero expression in the *bantam* mutant probably contributes to the reduction of INP numbers. We did not observe a change in Prospero levels in *bantam* mutant type I neuroblasts (Fig. S4B). The prolonged division of the type II lineage might allow more time for target expression to accumulate, compared with the more rapid progression of the type I lineage. However, other biologically interesting explanations have not been excluded.

### Brat and Prospero mediate the effects of bantam on neuroblast growth and proliferation

**Fig. 5.**
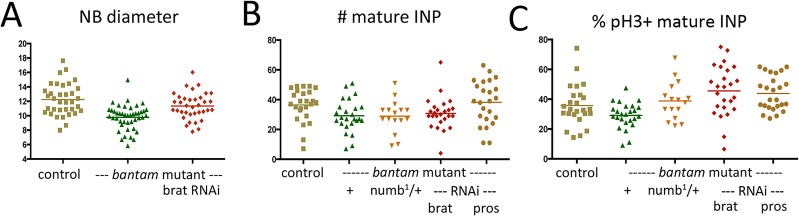
**Genetic evidence that bantam acts via regulation of *brat* and *prospero.*** (A-C) MARCM clonal analysis showing *bantam^Δ1/Δ1^* mutant clones expressing the indicated UAS-RNAi transgenes. (A) Type II NB diameter (µm) in control FRT2A clones, *bantam^Δ1/Δ1^* mutant clones and *bantam^Δ1/Δ1^* mutant clones expressing *UAS-brat RNAi*. Control: *n*=35; *bantam* mutant *n*=42; with *brat* RNAi *n*=36 clones. ANOVA: *P*<0.0001 comparing control and *bantam* mutant; *P*=0.0008 comparing *bantam* mutant with and without *brat* RNAi. The control and *bantam* mutant samples are the same as those shown in Fig. 3E. NB diameter was not measured in the *brat* RNAi control clones in an otherwise wild-type background. (B) Total number of mature INPs (Dpn^+^Ase^+^) in type II NB clones of the indicated genotypes. *n*=25 clones for the control, *bantam^Δ1/Δ1^* mutant and *bantam* mutant with *brat* RNAi. *n*=34 clones for the *bantam* mutant with *prospero* RNAi and *n*=16 with *numb^1^.* ANOVA: *P*=0.029 comparing *bantam* mutant with and without *prospero* RNAi. The other comparisons were not significant. The control and *bantam* mutant samples are the same as those shown in Fig. 3G. Each clone contained a single large Dpn^+^Ase^−^ NB, so the change in clone size and INP number/clone cannot be attributed to an increase in NB number, as occurs in *brat* or *prospero* mutant brains. (C) Percentage of mature INPs labeled with anti-pH3 in type II NB clones of the indicated genotypes. *n*=25 clones for the control, *bantam^Δ1/Δ1^* mutant and *bantam* mutant with *brat* or *prospero* RNAi and *n*=16 with *numb^1^*. ANOVA: *P*<0.0001 comparing *bantam* mutant with and without *brat* RNAi and *P*=0.0005 comparing *bantam* mutant with/without *prospero* RNAi. The effect of removing one copy of *numb* was not statistically significant by ANOVA (*P*=0.07, comparing all samples in the experiment), but was significant in a pairwise comparison of the *bantam* mutant with and without *numb* RNAi using an unpaired *t*-test (*P*=0.013 assuming unequal variance). The control and *bantam* mutant samples are the same as those shown in Fig. 3J.

To assess the impact of increased target expression in the type II lineage, we used *Elav-Gal4* to express UAS-RNAi transgenes to lower their expression in the *bantam* mutant MARCM clones. Selectively depleting individual targets in *bantam* mutant clones allows a direct test of whether their elevated expression contributes to the mutant phenotype. UAS-RNAi mediated depletion of *brat* transcript significantly increased the average size of *bantam* type II neuroblasts (Fig. 5A). Selective depletion of *prospero* was sufficient to restore the number of mature INP cells in the *bantam* mutant clones (Fig. 5B), whereas depletion of *brat* or removal of one functional copy of *numb* had no significant effect on mature INP number (Fig. 5B). Selective depletion of *brat* or *prospero* resulted in a significant increase in the proportion of pH3^+^ mitotic INPs in *bantam* mutant clones (Fig. 5C; representative images are in Fig. S8). Increased INP proliferation probably contributes to suppression of the *bantam* mutant phenotype. However, this does not exclude the possibility that there might also be some dedifferentiation of GMCs to produce more INPs in the *prospero*-depleted condition.

Removing *brat*, *prospero* or *numb* gene function can lead to overproliferation and, in some cases, to tumor formation (Li and Vaessin, 2000; Betschinger et al., 2006; Choksi et al., 2006; Bello et al., 2008). The *numb* experiments used the *numb^1^* mutant allele, which does not cause neuroblast overproliferation as a heterozygote. To control for the effects of depleting *brat* and *prospero*, we made MARCM clones to express the UAS-RNAi transgenes in an otherwise wild-type background. The number of GFP^+^ progeny (clone size) and the number of Dpn^+^Ase^+^ mature INPs per clone did not increase under these conditions (Fig. S8). Thus, the level of *brat* and *prospero* downregulation that was achieved in the MARCM clones was not sufficient to cause INP amplification on its own. On this basis, we conclude that the RNAi rescue worked by offsetting the increase in transcript levels in the *bantam* mutant.

Taken together, these findings provide evidence that misregulation of *brat* and *prospero*, and perhaps also *numb*, contributes to the consequences of removing bantam activity from the type II neuroblast lineage. They provide evidence that upregulation of *brat* contributes to the growth defect in *bantam* mutant neuroblasts. These findings are consistent with the known role of Brat as an inhibitor of type II NB cell growth (Sonoda and Wharton, 2001; Bowman et al., 2008; Betschinger et al., 2006; Bello et al., 2008). Similarly, they provide evidence that upregulation of *prospero* contributes to the reduced proliferation of *bantam* mutant clones, consistent with the later role of Prospero to limit neuroblast proliferation and to promote differentiation (Li and Vaessin, 2000; Choksi et al., 2006). The effects of the *bantam* mutant, mediated via misregulation of *prospero* are consistent with an earlier report showing that Prospero overexpression can suppress INP proliferation (Bayraktar et al., 2010). The evidence supporting contributions of *brat* and *numb* overexpression to INP number proliferation are less clear-cut. Depletion of *brat* had a significant effect on the number of proliferating cells detected by the pH3 label, but did not significantly alter total INP number. Reduced growth might be indirectly responsible for the effect on INP proliferation, but we do not rule out the possibility that Brat might also act in other ways to limit INP proliferation. Numb appears to have some effect on INP proliferation, albeit not quite reaching the level of statistical significance.

### Post-transcriptional control of Prospero in the type II lineage

Although type I neuroblasts far outnumber type II neuroblasts, each type II neuroblast contributes a large population of neurons to the adult *Drosophila* brain as a result of INP transit amplification. Differential expression of the differentiation factor Prospero in the immediate progeny of the neuroblasts is a key difference between the type I and II lineages. Prospero is nuclear in type I GMCs, but this is suppressed in the type II INPs, which allows the INPs to undergo further rounds of asymmetric division. Consequently, each INP makes several neuronal progeny rather than two for each type I GMC (Bayraktar et al., 2010).

Our studies provide evidence for a new post-transcriptional layer of control of Prospero in the type I and II lineages. Previously, work has shown a role for the zinc finger transcription factor Earmuff in the transcriptional control of *prospero* expression in maturing INPs, thereby limiting their proliferation and promoting terminal differentiation (Weng et al., 2010). Our findings provide evidence that bantam miRNA limits premature Prospero expression in the type II lineage*.* The increased level of *prospero* transcript in the *bantam* mutant neuroblast lineages presumably leads to premature nuclear accumulation of Prospero protein in INP cells. Thus, *prospero* appears to be under both transcriptional and post-transcriptional control in the type II lineage. The effect of bantam on regulation of *prospero* expression appears to be direct, based on comparing intact and mutant versions of the *prospero* 3′ UTR in luciferase reporter assays (Fig. S9).

The type II neuroblast lineage appears to be more prone to tumor formation. Loss of the Notch pathway repressor Numb or the translational repressor Brat results in tumor formation in type II lineages, but not in type I lineages, although both types of neuroblast express these factors (Bello et al., 2008; Bowman et al., 2008). Loss of Earmuff in the type II lineage also causes tumors (Weng et al., 2010). Tumor formation is likely to be a consequence of continued proliferation of the INPs, and may involve reversion towards a type II neuroblast identity. As might be expected, based on its role in supporting INP expansion and proliferation, removing bantam activity was able to partially offset the effects of depleting *brat* by RNAi selectively in type II lineages using the *wor-Gal4 ase-Gal80* combination (Fig. S10)*.* It will be of interest to learn whether misregulation of miRNAs that confer post-transcriptional regulation of other stem-cell proliferation and differentiation regulators have roles in CNS tumor formation. In this context, it is interesting that bantam activity is required for the formation of ovarian tumors resulting from removal of the Brat-related TRIM-NHL protein Mei-P26 (Neumüller et al., 2008).

## MATERIALS AND METHODS

### Fly stocks

Flies were maintained on standard yeast-cornmeal-agar medium at 25°C unless otherwise stated. *bantam^Δ1^*, bantam sensor and *UAS*-*bantam* are described by Brennecke et al. (2003) and *UAS*-*bantam-3p* sponge in (Becam et al., 2011); UAS-DIAP1 is described by Wang et al. (1999). *UAS*-*prospero*-*RNAi* and *UAS*-*brat*-*RNAi* lines were from Vienna *Drosophila* RNAi Center (#101477 and #105054). Elav-Gal4, UAS-redStinger, *pros^17^* and *numb^1^* were from Bloomington *Drosophila* Stock Center (#458, #8546, #5458 and #4096, respectively). *bantam-lacZ* (P{lacW}banL1170a) is described by Hipfner et al. (2002). *insc*-*Gal4* is described by Betschinger et al. (2006). *worGal4, aseGal80/Cyo* was used to drive expression in type II NB clones (Neumüller et al., 2011). *w^1118^* flies were used as the wild-type control unless otherwise stated.

### MARCM analysis

To generate positively labeled MARCM clones, *hsFLP, elav-Gal4; UAS-mCD8::GFP, UAS-lacZ/ CyO; FRT2A, tubP-Gal80/TM6b* were crossed to *FRT2A* or *FRT2A, bantam^Δ1^/TM6b* or *UAS*-*pros*-*RNAi/UAS*-*pros*-*RNAi*; *FRT2A, bantam^Δ1^/TM6b* or *UAS*-*brat*-*RNAi*/*UAS*-*brat*-*RNAi*; *FRT2A, bantam^Δ1^/TM6b* or *numb^1^/CyOKrGFP; FRT2A, bantam^Δ1^/TM6b* or UAS-ban^A^/CyOKrGFP; *FRT2A, bantam^Δ1^/TM6b*. Embryos were collected over 4-6 h, and raised at 25°C for 21-25 h before a 45 min heat shock at 37°C. Larvae were then raised at 25°C until dissection at 120 h ALH.

### TU tagging

TU tagging was performed as described (Miller et al., 2009), with the following modification. Larvae of the indicated genotypes were collected in groups of 20 at 72 h ALH, transferred to food vials with 4-thiouracil (4-TU)-containing yeast paste at 25°C for 16 h before CNS dissection. For subsequent experiments, 170 dissected CNS tissues from the following genotypes were used: inscGal4>UAS-UPRT2.1-HA; Dr or TM2/+ (control) vs inscGal4>UAS-UPRT2.1-HA; *bantam^Δ1/Δ1^* (mutant).

### RNA analysis

For quantitative real-time RT-PCR, total RNA was purified with TriZol. For the quantification of protein-coding genes, total RNA samples were treated with on-column DNase for 60 min at room temperature to eliminate DNA contamination (Qiagen), and first strand synthesis used oligo-dT primers and SuperScript RT-III (Invitrogen). Measurements were normalized to *rp49* (*RpL32* – FlyBase). For quantification of miRNA mature transcript levels, reverse transcription reactions were performed using the TaqMan microRNA Reverse Transcription Kit (Life Technologies). qPCR was performed using the Taqman Universal PCR Mastermix (Life Technologies). Measurements of *bantam* mature transcript level were normalized to miR-2b and miR-184.

### Immunocytochemistry and imaging

Larval and adult brains were dissected and fixed in 4% formaldehyde in PBS with 0.2% Triton X-100 for 20 min at room temperature. For larval brains, samples were incubated with primary antibodies at 4°C overnight and secondary antibodies at room temperature for 2 h. For adult brains, samples were incubated with primary antibodies at 4°C for 2 nights and secondary antibodies at room temperature for 2 h. The following primary antibodies were used at the indicated dilutions: rat anti-Elav [Developmental Studies Hybridoma Bank (DSHB), 7E8A10; 1:50], mouse anti-Prospero (DSHB, MR1A; 1:10), mouse nc-82 (DSHB; 1:20), rabbit anti-phosphohistone H3 (Cell Signaling, 9701S; 1:200), chicken anti-GFP (Abcam, 13970; 1:2000), rabbit anti-Ase (Brand et al., 1993) (1:200), guinea pig anti-Deadpan (provided by James Skeath, Department of Genetics, Washington University of St. Louis; 1:2000) and anti-Eyeless (Kammermeier et al., 2001) (1:200). Secondary antibodies were Alexa Fluor 405-, Alexa Fluor 555-, Alexa Fluor 633-, or Alexa Fluor 488-conjugated (Invitrogen, A-11039, A-21435, A-21105, A-21072, A-21094, A-21200) and used at 1:300, 1:500, 1:300 and 1:500, respectively. DNA stain was DAPI (Sigma). Samples were mounted in Vectashield. Quantification was performed using ImageJ and Imaris (Bitplane).

### Cell transfection and luciferase assays

S2 cells were transfected in 24-well plates with 250 ng of pBS-Actin-Gal4, 125 ng of *pUAST-dsRed*-*bantam-3p* or -*5p* sponge plasmid or empty pUAST-dsRed vector, 25 ng of firefly luciferase reporter plasmid, and 25 ng of *Renilla* luciferase DNA as a transfection control. Transfections were performed in triplicate in at least three independent experiments. Dual luciferase assays (Promega) were performed 72 h after transfection according to the manufacturer's instructions. Luciferase activity was normalized to total protein content, measured on the same sample using the Bradford method (Bio-Rad). Where necessary, 60 µl of cell lysate was added to 940 µl of TriZol for total RNA extraction. *pros* 3′UTR sequence (partial) was PCR amplified from genomic DNA from canton-S flies using oligos 5′-ACCGCTCGAGGAGCCGTAAACGTAAGCAACCG-3′ (forward) and 5′-ACCGCTCGAGGTCTGCTTTTCAACGGGAAATAAGATTA-3′ (reverse) and subcloned (*Xho*I/*Xho*I) after luciferase, under the control of tubulin promoter as described by Brennecke et al. (2003). *pros* UTR reporters with mutated *bantam* 5p or 3p sites were produced by PCR using primers designed to change both the seed region and 3′ region. The reporter constructs were sequence verified. The *bantam* 5p sponge sequence [GCGGCCGCA(AGTCAAACCAATACAAAACCGGGATA)_9_AGTCAAACCAATACAAAACCGGTCTAGA] contains ten binding sites that are complementary to the mature *bantam* sequence and has a central bulge to prevent direct mRNA cleavage. The DNA sequence was commercially synthesized, and subcloned (*Not*I/*Xba*I) downstream of a dsRed coding sequence into a pUAST vector.
